# Comparison of the Performances of Different Computational Methods to Calculate the Electrochemical Stability of Selected Ionic Liquids

**DOI:** 10.3390/ma14123221

**Published:** 2021-06-10

**Authors:** Annalisa Paolone, Sergio Brutti

**Affiliations:** 1 Consiglio Nazionale delle Ricerche, Istituto dei Sistemi Complessi, Piazzale Aldo Moro 5, 00185 Rome, Italy; sergio.brutti@uniroma1.it; 2 Department of Chemistry, Sapienza University of Rome, Piazzale Aldo Moro 5, 00185 Rome, Italy; 3 GISEL—Centro di Riferimento Nazionale per i Sistemi di Accumulo Elettrochimico di Energia, INSTM via G. Giusti 9, 50121 Firenze, Italy

**Keywords:** ionic liquids, density functional theory, electronic structure, electrochemical stability window, imide anions

## Abstract

The electrochemical stability windows (ESW) of selected ionic liquids have been calculated by comparing different computational approaches previously suggested in the literature. The molecular systems under study are based on di-alkyl imidazolium and tetra-alkyl ammonium cations coupled with two different imide anions (namely, bis-fluorosulfonyl imide and bis-trifluoromethyl sulfonyl imide), for which an experimental investigation of the ESW is available. Thermodynamic oxidation and reduction potentials have here been estimated by different models based on calculations either on single ions or on ionic couples. Various Density Functional Theory (DFT) functionals (MP2, B3LYP, B3LYP including a polarizable medium and empirical dispersion forces) were exploited. Both vertical and adiabatic transitions between the starting states and the oxidized or reduced states were considered. The approach based on calculations on ionic couples is not able to reproduce the experimental data, whatever the used DFT functional. The best quantitative agreement is obtained by calculations on single ions when the MP2 functional in vacuum is considered and the transitions between differently charged states are vertical (purely electronic without the relaxation of the structure). The B3LYP functional underestimates the ESW. The inclusion of a polar medium excessively widens the ESW, while a large shrinkage of the ESW is obtained by adopting an adiabatic transition scheme instead of a vertical transition one.

## 1. Introduction

Electrochemical devices, such as lithium batteries, fuel cells and solar cells, require suitable electrolytes able to allow a facile migration of ions while being inert at the electrodes. Electrolytes must fulfill many requirements: high ionic conductivity, high thermal and electrochemical stability and wide liquid ranges, among the others. Under these aspects, many ionic liquids (ILs) are apparently suitable to compete and replace standard organic solvents, thanks to their specific physico-chemical properties [1,2]. With the development of high voltage lithium batteries, the need for innovative electrolytes able to sustain highly oxidizing electric potentials is becoming more apparent and poses new challenges.

Experimentally, many ionic liquids have been investigated to establish their electrochemical stability window [3,4]. Overall, the anions of the ionic liquids play a central role, as they inevitably determine the highest voltage at which the devices can operate without the degradation of the electrolyte (anodic limit) [4]. However, from an experimental point of view, the determination of the electrochemical stability of ionic liquids poses some challenges and the interpretation of data is not always straightforward. Indeed, it is well known that the chemical nature of the electrodes (e.g., Pt, Ni, stainless steel, aluminum, carbon composites, etc.) can increase or decrease the electrochemical stability window; moreover, the acceptable current level for the definition of non-degrading electrolyte varies from study to study [4], as well as between data analysis algorithms. Finally, different reference electrodes are often used for different applications, thus shifting the anodic and cathodic ranges. In general, however, it has been shown that the ionic liquids with the highest anodic stability are highly fluorinated.

The electron affinity and ionization of anions and cations composing ionic liquids have also been investigated computationally by many authors, and some correlation between computational results and the electrochemical stability window (ESW) has been proposed. A simple approach suggests a linear correlation between the energy of the highest unoccupied molecular orbital (HOMO) of anions and their oxidation potential [5,6]. On the other hand, the computation of the energy difference for a vertical transition from the charged ion and the neutral state is easily performed, either in the gas phase or in the presence of a suitable polarizable medium (PM). This second approach allows us to mimic ionic liquids’ oxidation and reduction in liquid media [7,8]. Overall, the inclusion of the PM strongly overestimated the electrochemical stability while, in the gas phase, DFT calculations with the B3LYP or VSXC functional provided results in quantitative agreement with experimental data [7]. Computational studies have also been carried out on cations, and a detailed investigation of the changes of their reduction potential in respect to substitutions in their alkyl chain has been reported, exploiting hybrid DFT with the “classic” B3LYP functional [9]. Furthermore, the linear correlation between electron affinities estimated through vertical and adiabatic transitions between charged and neutral states hasbeen sketched [9]. A combination of DFT calculations and molecular dynamics was used by Ong et al. [10] to estimate the ESW of neutral ionic pairs between common cations (1-butyl-3-methylimidazolium (BMIM) and N,N-propylmethylpyrrolidinium (P_13_)) and simple anions, PF_6_, BF_4_, and bis(trifluoromethylsulfonyl)imide (TFSI). Additionally, ab-initio molecular dynamics have been proposed for the investigation of ESW [11]. Various DFT functionals have been applied to establish a correlation between HOMO-LUMO energy differences, ionization potentials and electron affinities for both adiabatic or vertical transitions of single ions or ion pairs in various chemical systems [12,13,14]. Cheng [15] and Kazemiabnavi [16] exploited full thermodynamic cycles to calculate the reduction and oxidation potentials of ILs, following a strategy suggested by Winget et al. and Marenich et al. for simple systems [17,18]. Cheng and Kemiabnavi modelled the thermodynamics of oxidation and reduction processes, also considering ion salvation [15,16]. Similar approaches have also been used for a high throughput screening of hundreds of possible anions and cations obtained by substitutions with defined functional groups in common anions and cations [15,19], using hybrid-DFT calculations and the B3LYP functional.

**Figure 1:**
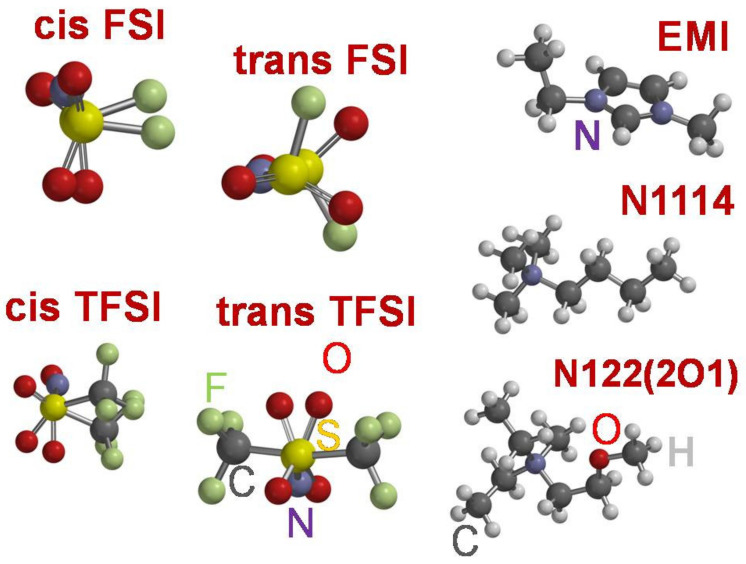
Schematics of the anion and cations investigated in the present study.

We recently reported an experimental physico-chemical investigation of novel liquids based on imidazolium or tetra-alkyl-ammonium cations, coupled with bis(perfluroalkylsulfonyl)imide anions (i.e., EMIFSI, EMITFSI, N1114FSI, N1114TFSI and N122(2O1)TFSI, respectively). We demonstrated their use as solvents in electrolytes for lithium-ion batteries working up to 5 V [20,21]. The anodic stability of these ionic liquids was measured on carbon composite electrodes over aluminum using a 0.8:0.2 molar mixture with the LiTFSI salt [20] and, in all cases, they overcame 4.5 V vs. Li. Here, we tackle the challenge of a systematic computational investigation of the thermodynamic stability widows of these ionic liquids by comparing the experimental data to various computational approaches proposed in the literature. Our aim is to evaluate the reliable computational approximation for a precise estimate of the ESWs of ionic liquids. We carried out this study on the five different ionic liquids studied by us previously [20]; the structure of the ILs ionic constituents is shown in Figure 1.

## 2. Materials and Methods

All computational investigations of the single ions or of the ionic couples composing the ionic liquids were performed by means of Spartan software [22]. For each ion or ionic couple, possible conformers were investigated by means of a systematic rotation of flexible bonds, exploiting built-in-routines. Unrelaxed and relaxed structures were computed at MP2 [23] or DFT levels of theory [24]: 0K electronic energy as well as Gibbs energy at finite temperatures considering thermal effects were calculated. Various computational methods were used: MP2 [25,26], B3LYP [27], B3LYP with a polar solvent (dimethylformamide, ε_r_ = 37.22) using the C-PCM algorithm [28] and B3LYP with the same polar solvent and dispersion forces. For all calculations, a 6-31G** basis set was employed.

Oxidation and reduction reactions were mimicked using different approaches, starting from a structurally relaxed minimum energy molecule/ion (initial state):

1. Vertical transition of single ions, where the final state preserves the structure of the initial state.
2. Adiabatic transition of single ions, where the final state is energetically relaxed to its minimum.
3. Vertical or adiabatic transition of neutral ionic couples.

In the case of vertical transitions, ESW were estimated, starting from oxidation/reduction reaction energies calculated by subtracting the computed total energy, i.e., *E_tot_*, of the final state (product) to that of the initial one (reagent). For an oxidation reaction where the reagent (initial state) is the chemical specie A with net charge n:

(1) $$\begin{array}{c} A^{n}\to A^{n-1}+e^{-}\\ \;\;\;\;\;\;\Delta E_{anodic}=E_{tot}\left(A^{n}\right)-E_{tot}\left(A^{n-1}\right) \end{array}$$

4. For a reduction reaction where the reagent (initial state) is the chemical specie B with net charge *m*:

(2) $$\begin{array}{c} B^{m}+e^{-}\to B^{m+1}\\ \;\;\;\;\;\;\;\Delta E_{cathodic}=E_{tot}\left(B^{m}\right)-E_{tot}\left(B^{m+1}\right) \end{array}$$

For anions, i.e., *A*^−^, the oxidation reactions involve the transition from the anion to a neutral state, while the reduction reaction is due to the transition from the anion to a dianion state. The first reactions give the anodic limit, while the second is responsible for the cathodic one.

(3) $$\begin{array}{c} A^{-}\to A+e^{-}\\ \;\;\;\;\;\;\Delta E_{Anodic}=E_{tot}\left(A\right)-E_{tot}\left(A^{-}\right) \end{array}$$

(4) $$\begin{array}{c} A^{-}+e^{-}\to A^{2-}\\ \;\;\;\;\;\;\;\;\Delta E_{Cathodic}=E_{tot}\left(A^{2-}\right)-E_{tot}\left(A^{-}\right) \end{array}$$

For cations, the oxidation transforms the cation into a di-cation, and it is responsible for anodic stability, while the reduction transforms the cation into a neutral species and is linked to the cathodic limit.

(5) $$\begin{array}{c} B^{+}\to B^{2+}+e^{-}\\ \;\;\;\;\;\;\Delta E_{Anodic}=E_{tot}\left(B^{2+}\right)-E_{tot}\left(B^{+}\right) \end{array}$$

(6) $$\begin{array}{c} B^{+}+e^{-}\to B\\ \;\;\;\;\;\;\;\;\Delta E_{Cathodic}=E_{tot}\left(B\right)-E_{tot}\left(B^{+}\right) \end{array}$$

In the case of adiabatic transitions, either starting from ions or ionic couples, the energy differences can be calculated by considering explicitly zero-point energies (ZPE) for both reagents and products, both of which have vibration real frequency values. Thus, one may obtain the $\Delta G_{0K,anodic}^{o}$ and the $\Delta G_{0K,cathodic}^{o}$ starting from the Gibbs energies at 0K for the initial and final states (namely $G_{tot,0K}=E_{tot}+ZPE)$.

The representation of ESW using the usual relative electrochemical potentials has been obtained in the case of vertical transitions by the following relations:

(7) Anodic limit V vs. Li=ΔEAnodicF−1.46

(8) Cathodic limit V vs. Li=−ΔECathodicF−1.46

where *F* is the Faraday constant and ΔE is the oxidation or reduction electronic reaction energy, and the term−1.46 V is due to the necessity of referring these limits to the standard Li^+^/Li^0^ electrode, as discussed in ref. [12]. In the case of adiabatic transitions, the ΔE is substituted by $\Delta G_{0K}^{o}$.

## 3. Results

### 3.1. Vertical Transitions Approximation Starting from Ions

Table 1, Table 2, Table 3 and Table 4 report the electronic energy of the ions and neutral species for the two conformers of the FSI and TFSI anions, and for the lowest energy conformer of the cations; the corresponding anodic and cathodic limits, calculated according to Equations (7) and (8), are also reported. Each table comprises a specific computational method:

1. MP2 in vacuum.
2. B3LYP in vacuum.
3. B3LYP in a simulated polar medium
4. B3LYP in a simulated polar medium with the inclusion of empirical dispersion forces.

**Table 1:** Electronic energy (in atomic units) of anions and cations and their unrelaxed neutral or double charged forms calculated at the MP2/6-31G** level of theory and derived anodic and cathodic limit (V).

**Anion**	***E_tot_*(*A*^−^)**	***E_tot_*(*A*)**	**Anodic** **Limit**	***E_tot_*(*A*^2−^)**	**Cathodic Limit**
trans TFSI	−1823.63092	−1823.39173	4.99	−1823.34804	−9.08
cis TFSI	−1823.63007	−1823.39515	4.87	−1823.33703	−9.36
transFSI	−1349.23486	−1348.99802	4.92	−1348.94736	−9.21
cisFSI	−1349.23324	−1349.00147	4.79	−1348.94942	−9.11
**Cation**	***E_tot_*(*B*^+^)**	***E_tot_*(*B*)**	**Cathodic Limit**	***E_tot_*(*B*^2+^)**	**Anodic Limit**
N1114	−331.02025	−331.04434	−0.81	−330.45111	13.88
N122(2O1)	−445.22449	−445.22282	−1.50	−444.71155	12.36
EMI	−343.49078	−343.56614	0.57	−342.96762	12.64

**Table 2:** Electronic energy (in atomic units) of anions and cations and their unrelaxed neutral or double charged forms calculated at the B3LYP/6−31G** level of theory and derived anodic and cathodic limits (V).

**Anion**	***E_tot_*(*A*^−^)**	***E_tot_*(*A*)**	**Anodic** **Limit**	***E_tot_*(*A*^2−^)**	**Cathodic Limit**
trans TFSI	−1827.20535	−1826.99235	4.28	−1826.95785	−8.13
cis TFSI	−1827.20097	−1827.00271	3.88	−1826.95553	−8.07
transFSI	−1351.66082	−1351.43819	4.54	−1351.40995	−8.22
cisFSI	−1351.6592	−1351.44255	4.38	−1351.41302	−8.09
**Cation**	***E_tot_*(*B*^+^)**	***E_tot_*(*B*)**	**Cathodic Limit**	***E_tot_*(*B*^2+^)**	**Anodic Limit**
N1114	−332.13521	−332.18441	−0.13	−331.58873	13.26
N122(2O1)	−446.65622	−446.68747	−0.61	−446.16199	11.85
EMI	−344.56568	−344.66596	1.25	−344.04875	12.47

**Table 3:** Electronic energy (in atomic units) of anions and cations and their unrelaxed neutral or double charged forms calculated at the B3LYP/6-31G** level of theory in a polar medium and derived anodic and cathodic limit (V).

**Anion**	***E_tot_*(*A*^−^)**	***E_tot_*(*A*)**	**Anodic** **Limit**	***E_tot_*(*A*^2−^)**	**Cathodic Limit**
trans TFSI	−1827.28312	−1827.00165	6.13	−1827.24442	−2.50
cis TFSI	−1827.28187	−1827.00041	6.12	−1827.24039	−2.58
transFSI	−1351.73851	−1351.44738	6.39	−1351.71605	−2.07
cisFSI	−1351.73742	−1351.45178	6.24	−1351.71803	−1.98
**Cation**	***E_tot_*(*B*^+^)**	***E_tot_*(*B*)**	**Cathodic Limit**	***E_tot_*(*B*^2+^)**	**Anodic Limit**
N1114	−332.21925	−332.18622	−2.35	−331.88683	7.50
N122(2O1)	−446.73390	−446.69196	−2.59	−446.45716	6.00
EMI	−344.64676	−344.67272	−0.76	−344.36680	6.08

**Table 4:** Electronic energy (in atomic units) of anions and cations and their unrelaxed neutral or double charged forms calculated at the B3LYP/6-31G** level of theory, including empirical dispersion forces in a polar medium and derived anodic and cathodic limit (V).

**Anion**	***E_tot_*(*A*^−^)**	***E_tot_*(*A*)**	**Anodic** **Limit**	***E_tot_*(*A*^2−^)**	**Cathodic Limit**
trans TFSI	−1827.30034	−1827.01849	6.14	−1827.25980	−2.55
cis TFSI	−1827.29967	−1827.01740	6.15	−1827.25726	−2.60
transFSI	−1351.74681	−1351.45603	6.38	−1351.72454	−2.06
cisFSI	−1351.74565	−1351.46024	6.23	−1351.72263	−2.08
**Cation**	***E_tot_*(*B*^+^)**	***E_tot_*(*B*)**	**Cathodic Limit**	***E_tot_*(*B*^2+^)**	**Anodic Limit**
N1114	−332.24174	−332.20839	−2.36	−331.90927	7.50
N122(2O1)	−446.76405	−446.72115	−2.62	−446.48594	6.03
EMI	−344.65858	−344.68410	−0.77	−344.37864	6.08

In all cases, the structure of the initial anion or cation was optimized at the corresponding level of theory.

In all cases, as expected, the anodic and cathodic limit of the anion is lower than that of the cation; therefore, the limits of stability of whole ionic liquid are the anodic limit of the anion and the cathodic limit of the cation.

The MP2 method (see Table 1) gives anodic limits of the conformers for the two bis(perfluroalkylsulfonyl)imide anions values between 4.8 and 5.0 V and estimates in the range of −1.5 to 0.5 V for the cathodic limit of the cations. These values are in good agreement with our experimental determinations [20].

The electrochemical stability window shrinks slightly while using the B3LYP functional: the anodic limit decreases to 3.9/4.5 V (Table 2) while the cathodic limit increases around −0.6/+1.2 V. These values slightly underestimate the oxidation limit while overestimating the reduction stability. The introduction of a polar medium widens the electrochemical stability window (−2.4/+6.4 V) (see Table 3), overcoming the experimental determinations, while the addition of empirical dispersion forces has a marginal effect (see Table 4).

### 3.2. Adiabatic Transition Approximation Starting from Ions

Table 5, Table 6 and Table 7 report the values obtained at the levels of MP2, B3LYP in vacuum and B3LYP in a polar medium. Due to the increased computational time needed to calculate the vibrational properties of ions, we restricted calculations to the anodic limit of the anions and the cathodic limit of the cation; these are the relevant values to derive the electrochemical stability of the ionic liquids.

Compared to the figures obtained for vertical transitions, the adiabatic transitions values are lower for the anodic stability limit and higher for the cathodic limit. This effect is expected since the structural relaxation of reduction/oxidation products leads to a stabilization of the final state and thus to smaller reaction energies. Overall, the EWSs range between 2.1/2.3 V and 3.5/4.1 V for the MP2 model (see Table 5), 2.2/2.9 V and 3.2/3.6 V for the B3LYP functional (see Table 6) and between−0.1/+1.1 V and 5.1/5.6 V for B3LYP in a polar medium (see Table 7).

These values are clearly not consistent with the experimental ones [20]. Moreover, in the case of relaxed neutral EMI at the MP2 level and of N122(2O1) at the B3LYP level, no convergence of the structure could be obtained, even after more than 1000 iterations, due to the structural instability of both doublet final states. One should also note that after the adiabatic transitions, starting from ammonium ions, one of the alkyl chains tended to detach from the N atom, suggesting a tendency for cleavage by the molecule. It must be noted that the variation of ZPE between the initial and final states of the transition is small (<30 kJ/mol) in comparison to the electronic energy difference; therefore, ZPE practically cancels out upon subtraction.

**Figure 2:**
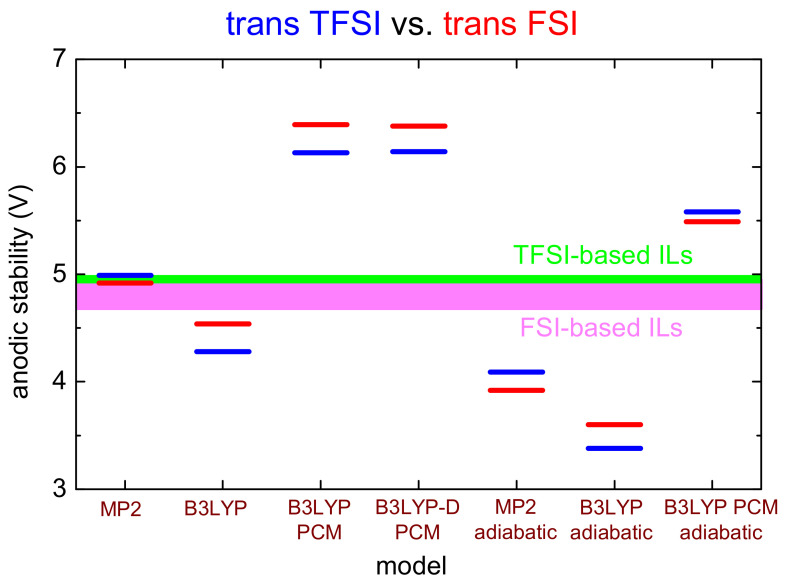
Comparison of the anodic limit calculated for trans-TFSI and trans-FSI with various theory levels, in vacuum or in a polarizable medium, considering a vertical or an adiabatic transition between differently charged states. The horizontal colored regions display the interval of values of the experimental anodic stability of the TFSI- and FSI-based ionic liquids investigated computationally in this paper.

To help to visualize the performances of various functionals in describing the ESW of TFSI and FSI anion, Figure 2 reports a graphical representation of the anodic limits calculated at different theory levels compared to the experimental values.

### 3.3. Vertical and Adiabatic Transition Approximations Starting from Ionic Couples

Table 8 and Table 9 report the electronic energy of the neutral, positive and negative ionic couple, as well as the derived anodic and cathodic limits calculated at the B3LYP level of theory in a vacuum (Table 8) or in a polar medium (Table 9), adopting either a vertical or an adiabatic approximation. Calculations based on the B3LYP functional in a vacuum clearly give an extremely high anodic limit and an extremely low cathodic limit, either considering or not considering any structural relaxation. Additionally, values obtained by the B3LYP model in a polar medium from vertical transitions display the same behavior. The estimated B3LYP in a polar medium considering the structural relaxation of the ionic couples (adiabatic approximation) is apparently closer to that in the experimental determination. To confirm the reliability of this approach (ionic couples in polar media under adiabatic transition approximation) beyond the five ionic couples, we calculated the anodic and cathodic limits of a quite different ionic liquid: Butyl methyl imidazolium chloride (BMIMCl). The ESW of BMIMCl was experimentally investigated in Ref. [29] using an Ag/Ag+ reference electrode. Taking into account the shift between the Li and Ag reference electrode, BMIMCl is expected to have anodic and cathodic limits at 3.38 V and 1.12 V, respectively, which are far from our predictions for ionic couples. Therefore, even considering the partial error compensation provided by the simultaneous adiabatic approximation and the adoption of a continuous polar medium, it is unlikely that any of the methods based on calculations on ionic couples can provide reliable values of anodic or cathodic limits of ionic liquids.

## 4. Discussion

From the previously reported data, it is quite evident that the adoption of a specific approximation, either in terms of computational conditions or thermodynamic approximation, has a remarkable impact on the accuracy and precision of ESW prediction.

The accuracy of the adopted methodology can be evaluated for the anodic limits (oxidation potentials) for all five ionic liquids by comparing the computational data with the experimental determination reported by us previously [20]. Two statistic quantities can be evaluated:

1. MAE: mean absolute error
  (9) $$MAE/Volt=\frac{\sum_{i=N1114FSI}^{EMIFSI}AL_{computational}-AL_{experimental}}{N}$$
2. MRE: mean relative error
  (10) $$MRE/\%=\frac{\sum_{i=N1114FSI}^{EMIFSI}\frac{AL_{computational}-AL_{experimental}}{AL_{experimental}}}{N}$$
  where *AL_computational_* and *AL_experimental_* are the anodic limits calculated or measured experimentally, respectively, whereas N = 5 is the number of ionic liquids considered in this investigation. In Table 10, the summary of the MEA and MRE are reported for the eleven different computational/thermodynamic models.

The experimental values of the anodic stability of the five ionic liquids here investigated were derived from the data of Ref. [20]. They were 4.68, 4.93, 4.95, 5.00, 4.90 V for EMI-FSI, N1114-FSI, EMI-TFSI, N1114-TFSI and N122(201)-TFSI, respectively.

It can be noted from Figure 2 that, for the estimate of the oxidation potentials of ionic liquids, the adoption of the MP2 method in vacuum mimicking a vertical transition from the anion largely overcomes all the other computation/methodological approaches in terms of accuracy. Lowering the level of theory from MP2 to B3LYP leads to large errors in the estimate of the oxidation potentials. Of course, the experimental anodic stability of different ionic liquids slightly depends on the cation; however, the spread of these values is extremely small when compared to the large differences introduced by the calculations with different computational methods, especially in the case of TFSI-based ILs (see Figure 2).

Overall, the accuracy of the MP2 predictions is surprising since the “vertical transition approximation from ion in vacuum” is crude compared to the complexity of a real case. From a physical point of view at the atomic scale, an irreversible oxidation of ionic liquid is a vertical ionization of an adsorbed anion at the electrolyte/electrode interface. Thus, the “vertical transition approximation from ion in vacuum” neglects two major real physical effects: (a) the partial solvation provided by the molecules/ions surrounding the reagent/products in proximity to the electrode, and (b) the ZPE of reagents and products. One may speculate that the partial compensation of solvation/ZPE from reagent/products results in precise predictions beyond expectations.

On the other hand, once the level of theory decreases from MP2 to B3LYP, accuracy drops. Furthermore, all the other adopted approximations fail in their attempts to compensate the worse electronic structure description modeled by the hybrid-DFT functional B3LYP compared to the MP2 perturbational method, although there is better mimicking of solvation and ZPE.

The partial solvation effect cannot be modeled by (a) adopting a continuous model solvent (polar media), (b) computing the entire ionic couple or (c) combining continuous model solvent and the ionic couple. In all approaches, reagents are too stabilized, thus leading to unrealistic large oxidation potentials.

Turning to the impact of the ZPE variations during the transition, one may consider that it is computationally feasible but with a weak physical meaning. In fact, whereas the ZPE of the reagent can be very easily and accurately computed, the neutral product of the vertical transition is not a minimum structure and has a large majority of imaginary vibrational frequencies. One possible way to circumvent this drawback is to mimic oxidation through an adiabatic transition. In fact, after structural relaxation, the oxidized product can be a vibrational minimum with all positive vibrational frequencies. However, this approach leads to an unrealistic stabilization of the electron energy of the oxidation product, thus leading to unrealistic small oxidation potentials.

One may note that the simultaneous occurrence of these two computational artifacts (i.e., overstabilization of the reagents by the continuous solvation model and the overstabilization of the products by following the adiabatic transition approach) can partially compensate each other. In fact, both the estimates of the oxidation potentials using adiabatic transitions in a continuous solvent from ions show errors of 9% or 0.45 V, which is smaller in comparison to the predictions from vertical transition from ions in vacuum at the same level of theory (B3LYP, i.e., −14%, −0.71 V).

## 5. Conclusions

The anodic and cathodic limits of selected ionic liquids were investigated computationally by means of various methods to compare them with the available experimental results. In previous literature, different techniques based on calculations on single ions, as well as on ionic couples, were proposed. In the present investigation, methods involving calculations on ionic couples either overestimate electrochemical stability or give values of the anodic and cathodic limits which tend to be independent of the specific couple. Techniques based on calculations on single ions seem to be more reliable, especially those based on vertical electronic transition computed with the MP2 functional. DFT methods relying on the B3LYP functional either underestimate the ESW, when calculations are performed in vacuum, or largely overestimate the ESW, when ions are placed in a polarizable medium. Overall, the calculations considering vertical electronic transitions in vacuum of the single ions using the MP2 theory are in the best agreement with the previous experiments. This fact opens new perspectives for future validation of the model for the electrochemical stability of other liquids already experimentally investigated, as well as for the screening of anions and cations that have not already been studied from an experimental point of view.

## Figures and Tables

**Table 5:** Gibbs energy (in atomic units) of anions and cations and their relaxed neutral forms calculated at the MP2/6-31G** level of theory and derived anodic or cathodic limit (V).

**Anion**	***E_tot_*(*A*^−^)**	***E_tot_*(*A*)**	**Anodic** **Limit**
trans TFSI	−1823.62028	−1823.41428	4.09
cis TFSI	−1823.61928	−1823.43472	3.51
transFSI	−1349.23911	−1349.03932	3.92
cisFSI	−1349.23863	−1349.04332	3.80
**Cation**	***E_tot_*(*B*^+^)**	***E_tot_*(*B*)**	**Cathodic Limit**
N1114	−330.80012	−330.93418	2.15
N122(2O1)	−444.97169	−445.11011	2.27
EMI	−343.35246	n.a.	n.a.

**Table 6:** Gibbs energy (in atomic units) of anions and cations and their relaxed neutral forms calculated at the B3LYP/6-31G** level of theory and derived anodic or cathodic limit (V).

**Anion**	***E_tot_*(*A*^−^)**	***E_tot_*(*A*)**	**Anodic** **Limit**
trans TFSI	−1827.19679	−1827.01722	3.38
cis TFSI	−1827.19244	−1827.02017	3.18
transFSI	−1351.66633	−1351.47866	3.60
cisFSI	−1351.66656	−1351.47947	3.58
**Cation**	***E_tot_*(*B*^+^)**	***E_tot_*(*B*)**	**Cathodic Limit**
N1114	−331.92069	−332.08220	2.89
N122(2O1)	−446.41160	n.a.	n.a.
EMI	−344.43057	−344.56575	2.18

**Table 7:** Gibbs energy (in atomic units) of anions and cations and their relaxed neutral forms calculated at the B3LYP/6-31G** level of theory in the presence of a polar medium and derived anodic or cathodic limit (V).

**Anion**	***E_tot_*(*A*^−^)**	***E_tot_*(*A*)**	**Anodic** **Limit**
trans TFSI	−1827.27619	−1827.01489	5.58
cis TFSI	−1827.27456	−1827.02984	5.13
transFSI	−1351.74524	−1351.48717	5.49
cisFSI	−1351.74472	−1351.48705	5.48
**Cation**	***E_tot_*(*B*^+^)**	***E_tot_*(*B*)**	**Cathodic Limit**
N1114	−332.00411	−332.08443	0.70
N122(2O1)	−446.48997	−446.58365	1.06
EMI	−344.52342	−344.57263	−0.13

**Table 8:** Electronic energy (in atomic units) of the neutral ionic couples and of the ionic couple with the addition or removal of an electron, without or with the possible relaxation of the geometry and derived cathodic and anodic limits (in V). All calculations were performed at the B3LYP/6-31G** level of theory in gas phase.

**Couple**	***E_tot_*(*AB*)**	***E_tot_*(*AB*^−^)**	***E_tot_*(*AB*^+^)**	**Cathodic Limit**	**Anodic Limit**
vertical approximation					
N1114-TFSI	−2159.46931	−2159.39488	−2159.14778	−3.46	7.20
N122(2O1)-TFSI	−2273.986794	−2273.89748	−2273.66706	−3.87	7.16
EMI-TFSI	−2171.90189	−2171.86766	−2171.57799	−2.38	7.26
N1114-FSI	−1683.921127	−1683.85372	−1683.57549	−3.27	7.85
EMI-FSI	−1696.354203	−1696.32878	−1696.01017	−2.14	7.81
adiabatic approximation					
N1114-TFSI	−2159.468312	−2159.49395	−2159.178994	−0.77	6.34
N122(2O1)-TFSI	−2273.986794	−2273.984858	−2273.697808	−1.51	6.33
EMI-TFSI	−2171.90189	−2171.906218	−2171.60822	−1.34	6.45
N1114-FSI	−1683.921127	−1683.95163	−1683.616591	−0.64	6.75
EMI-FSI	−1696.354203	−1696.363319	−1696.046943	−1.21	6.82

**Table 9:** Electronic energy (in atomic units) of the neutral ionic couples and of the ionic couple with the addition or removal of an electron, without or with the possible relaxation of the geometry and derived cathodic and anodic limits (in V). All calculations were performed at the B3LYP/6-31G** level of theory in a polar medium.

**Couple**	***E_tot_*(*AB*)**	***E_tot_*(*AB*^−^)**	***E_tot_*(*AB*^+^)**	**Cathodic Limit**	**Anodic Limit**
vertical approximation					
N1114-TFSI	−2159.56461	−2159.53508	−2159.27601	−2.26	6.32
N122(2O1)-TFSI	−2274.08905	−2274.06063	−2273.81845	−2.23	5.83
EMI-TFSI	−2171.98306	−2171.99998	−2171.71060	−1.01	5.88
N1114-FSI	−1684.00735	−1683.99813	−1683.70824	−1.71	6.60
EMI-FSI	−1696.42519	−1696.44125	−1696.15003	−1.03	5.95
BMIM-Cl	−883.67826	−883.69723	−883.43663	−0.94	5.05
adiabatic approximation					
N1114-TFSI	−2159.56461	−2159.64148	−2159.30582	0.61	5.51
N122(2O1)-TFSI	−2274.08905	−2274.16691	−2273.82620	0.64	5.62
EMI-TFSI	−2171.98306	−2172.02960	−2171.72324	−0.21	5.54
N1114-FSI	−1684.00735	−1684.10311	−1683.74163	1.12	5.70
EMI-FSI	−1696.42519	−1696.47219	−1696.16292	−0.19	5.61
BMIM-Cl	−883.678262	−883.726384	−883.446842	−0.16	4.78

**Table 10:** MAE/V and MRE/% calculated for the eleven different computational/thermodynamic models adopted.

Computational Method	MP2 in Vacuum	B3LYP in Vacuum	B3LYP in Polar Medium	B3LYP with D in Polar Medium
Vertical transitions from ions				
MAE	−0.07	−0.71	1.30	1.30
MRE	−1%	−14%	27%	27%
Adiabatic transitions from ions				
MAE	−1.21	−1.39	0.45	
MRE	−25%	−28%	9%	
Vertical transitions from ionic couples				
MAE		2.56	1.65	
MRE		53%	34%	
Adiabatic transitions from ionic couples				
MAE		1.22	0.70	
MRE		25%	14%	

## Data Availability

Data is contained within the article.
